# Oral Therapy for Infective Endocarditis: Where Do We Stand?

**DOI:** 10.3390/pathogens14121249

**Published:** 2025-12-06

**Authors:** Fatima Allaw, Maya Dagher, Hiba Saliba, Jana Khalil, Souha S. Kanj

**Affiliations:** 1 Department of Internal Medicine, The View Hospital, Al Qutaifiya, Qatar; fballaw@gmail.com; 2 College of Medicine, Qatar University, Doha P.O. Box 2713, Qatar; 3 Division of Infectious Diseases, Department of Medicine, University Hospitals Cleveland Medical Center, Case Western Reserve University, Cleveland, OH 44106, USA; maya-dagher@hotmail.com; 4 Division of Infectious Diseases, Department of Internal Medicine, Faculty of Medicine American, University of Beirut, Beirut 1107 2020, Lebanon; hs204@aub.edu.lb; 5 Department of Internal Medicine, Faculty of Medicine, American University of Beirut, Beirut 1107 2020, Lebanon; jk133@aub.edu.lb; 6 Center for Infectious Disease Research, Faculty of Medicine, American University of Beirut, Beirut 1107 2020, Lebanon

**Keywords:** infective endocarditis, oral antibiotic therapy, step-down oral therapy, POET trial

## Abstract

Standard therapy for infective endocarditis (IE) usually requires 4–6 weeks of intravenous (IV) antibiotics, ensuring sustained bactericidal concentrations, yet resulting in prolonged hospitalization and increased cost of care. These challenges have driven interest in oral antibiotic therapy (OAT) as a step-down strategy for selected, clinically stable patients. This review summarizes the clinical evidence and pharmacokinetic and pharmacodynamic (PK/PD) rationale and practical considerations supporting step-down OAT in IE. Antibiotics such as amoxicillin, fluoroquinolones, linezolid, and rifampicin have high bioavailability and maintain effective serum and tissue concentrations, and can be used as a safe transition from IV to oral therapy. The pivotal POET randomized controlled trial (RCT) demonstrated noninferiority of OAT compared with continued IV therapy in stable patients with left-sided IE caused by *Streptococcus* spp., *Enterococcus faecalis*, methicillin-susceptible *Staphylococcus aureus*, or coagulase-negative staphylococci. Further real-world studies and meta-analyses confirmed comparable efficacy and safety, with reduced catheter-related complications and shorter hospitalization length for patients receiving OAT. The latest European Society of Cardiology (ESC) guidelines have incorporated OAT regimens derived from the POET protocol for stable patients meeting specific criteria, while the American Heart Association guidelines have not yet been revised. Many areas remain uncertain, such as the optimal timing of transition to oral therapy; the ideal antibiotic combinations and dosing; and the applicability to complex cases such as methicillin-resistant *S. aureus* (MRSA), Gram-negative, or pediatric infections. Overall, clinical evidence supports OAT as a safe and effective alternative to prolonged IV therapy in selected patients with IE, highlighting a major step toward more individualized, patient-centered management.

## 1. Introduction

Infective endocarditis (IE) is the direct invasion of cardiac endothelium by bacteria or other microorganisms circulating in the bloodstream. The incidence of IE has increased globally over the past decades. Despite advances in diagnostics and management, IE remains associated with high morbidity and mortality [1]. Standard therapy for IE relies on prolonged intravenous (IV) antibiotic administration, typically for 4–6 weeks. Although effective, IV therapy, including outpatient IV antimicrobial therapy (OPAT), presents several challenges, such as high healthcare costs, adverse drug reactions, and complications related to IV access [2,3].

As with many other infectious diseases, the limitations of IV therapy in IE have prompted increasing interest in shifting to oral antibiotic therapy (OAT) as a step-down strategy for selected patients. OAT became attractive because of convenient outpatient management, reduced cost, fewer complications of IV access, and earlier hospital discharge. However, concerns remain regarding oral bioavailability with possibly unpredictable pharmacokinetics and pharmacodynamics (PK/PD) of certain drugs, and consistency of bactericidal activity.

Historically, the literature on OAT for IE was mostly limited to case series, observational studies, and PK data [4,5]. However, the pivotal POET trial published in 2019 was the first randomized controlled trial (RCT) to demonstrate that in carefully selected patients with stable left-sided IE, switching to OAT was noninferior to continued IV therapy in efficacy and safety [6]. This landmark study has triggered a paradigm shift toward reconsideration of treatment approaches.

This review discusses the rationale behind OAT in bacterial IE, summarizes the emerging evidence supporting its use, and outlines the current position of guidelines, existing gaps, and the need for future research.

## 2. Bioavailability and Pharmacokinetics

Endocardial vegetations are dense aggregates of fibrin and platelets embedding a high bacterial inoculum, which limits drug penetration and increases the risk of relapse if antibiotic exposure is suboptimal [7]. From that emerged the concept of the need for prolonged IV therapy for IE to allow time for antibiotics to achieve sustained bactericidal concentrations at the site of infection.

The PK/PD principles guiding antibiotic choice in IE include time-dependent killing, in which β-lactams are most effective when drug concentrations remain above the minimum inhibitory concetration (MIC) for a sufficient portion of the dosing interval, supporting strategies such as prolonged infusion or continuous infusion in severe illness [8]. With exposure over time, the area under the curve over the minimum inhibitory concentration (AUC/MIC) is also essential for drugs such as glycopeptides like vancomycin, daptomycin, or oxazolidinones such as linezolid, where an optimized AUC, particularly for enterococcal infections, is desirable [9]. Finally, concentration-dependent killing is used by aminoglycosides and fluoroquinolones and relies on high peak concentrations relative to the MIC of the organisms [10,11].

Scientific evidence largely supports the use of OAT to maintain bactericidal activity and reduce relapse, reflecting the same principles that guide IV therapy. PK/PD considerations, in particular, explain why certain antibiotic classes detailed below are prioritized in clinical studies evaluating OAT for IE.

### 2.1. Amoxicillin

Amoxicillin is widely used against enterococcal and streptococcal bacteremia as it has a better bioavailability than other oral penicillins. However, the oral dose cannot match the IV dose because absorption is saturable and dose-dependent: bioavailability falls from 100% at 375 mg to 55% at 3000 mg, with higher doses leading to significant unabsorbed drug and more side effects [12]. This limits the ability of oral amoxicillin to cover organisms with higher MICs seen in some IE cases. PK/PD studies show that standard oral regimens (e.g., 1000 mg 3 times daily) are effective only if the MIC ≤ 2 mg/L, while much higher doses (≥2500 mg 3 times daily) would be required at MIC of ≥8 mg/L [13]. The forthcoming RODEO 2 trial is evaluating a 2000 mg 3 times daily dosing for IE [14]. Similar concerns apply to amoxicillin/clavulanate, where even high doses are required to achieve PK/PD targets, and its use should be confined to pathogens with an MIC ≤ 1–2 mg/L [15].

### 2.2. Semi-Synthetic Penicillin

Semi-synthetic penicillins remain the standard therapy for methicillin-susceptible *Staphylococcus aureus* (MSSA) infections, including IE. However, they are characterized by a suboptimal bioavailability, ranging from 38 to 50% for dicloxacillin to around 30% for oxacillin [16,17]. Food markedly reduces their absorption, with a more than 40% for cloxacillin and oxacillin according to a recent meta-analysis [18]. They have short half-lives and, therefore, require frequent dosing (typically every 4–6 h) to maintain therapeutic levels, which is crucial for successful management of IE [19]. To achieve bactericidal activity in IE, drug concentrations should remain above the MIC for 60–70% of the dosing interval [15].

### 2.3. Fluoroquinolones

Fluoroquinolones are usually popular options for OAT for various infectious diseases, given their known high bioavailability and concentration-dependent bactericidal activity. However, these are not usually considered the first option due to the emergence of resistance during treatment of *S. aureus* infections. Moxifloxacin is considered one of the most potent fluoroquinolones against *S. aureus*, with standard 400 mg/day dosing achieving much higher exposure than levofloxacin or ciprofloxacin, though resistance may emerge if the MIC of the organism is >0.06–0.125 mg/L [11,20]. However, newer agents such as delafloxacin demonstrate substantially lower MICs against *S. aureus* (MIC_50_ 0.004 µg/mL), although delafloxacin has not been studied in IE [21]. Clinical studies showed the efficacy of ciprofloxacin + rifampicin for right-sided *S. aureus* IE in people who inject drugs [22,23]. Further studies are needed to evaluate the newer fluoroquinolones. For viridans-group streptococci, data on fluoroquinolone use for IE are largely limited to experimental models. In animal models of endocarditis, levofloxacin and trovafloxacin demonstrated limited activity but remained less effective than standard β-lactams, particularly when the MICs were elevated (≥1–2 mg/L) [24,25]. Levofloxacin was shown to be ineffective in certain streptococcal models despite adequate exposure [26]. More studies are needed to evaluate the PK/PD of fluoroquinolones against viridans-group streptococci to investigate their potential role in oral therapy.

### 2.4. Linezolid

Linezolid has excellent oral bioavailability with reliable tissue concentration; endocarditis case series and reviews report its use, mostly as salvage therapy, relying on its AUC/MIC profile [6,27]. It is typically prescribed as 600 mg twice daily for all patients, but this uniform dosing ignores major differences in drug clearance. Because linezolid is primarily metabolized in the liver and about 30% is renally excreted, patients with obesity or with renal or hepatic impairment can accumulate toxic levels, while others may receive subtherapeutic exposure. This explains the wide interindividual variability seen with therapeutic drug monitoring (TDM), where some patients develop early myelotoxicity, and others are underdosed [28]. Lowering the dose to 300 mg twice daily in the setting of renal impairment reduces the risk of thrombocytopenia without compromising efficacy, and similar adjustments are needed for severe hepatic dysfunction. Importantly, efficacy also drops when the organisms have higher MICs (≥2–4 mg/L) [29].

### 2.5. Rifampicin and Clindamycin

Rifampicin has a strong oral absorption and strong biofilm penetration, making it a desirable option in combination regimens, particularly in prosthetic valve or device-associated endocarditis [6]. Oral clindamycin has been used for bacteremia but not studied in IE; however, dosing should be adjusted according to body weight and drug clearance. An important consideration is that oral co-administration with rifampicin should be avoided unless TDM is available, as rifampicin markedly reduces clindamycin exposure by lowering bioavailability from 60% to 15%. This interaction, however, is not observed when clindamycin is administered as a continuous IV infusion [11].

### 2.6. Trimethoprim–Sulfamethoxazole (TMP/SMX)

TMP/SMX is an appealing oral option due to its excellent bioavailability (up to 90%) and its good tissue penetration, including into vegetations. The standard dose of 160/800 mg twice daily maintains serum levels above the MIC for susceptible *S. aureus* strains [30,31]. TMP/SMX works synergistically by interfering with consecutive steps of folate synthesis, resulting in bactericidal activity against *S. aureus*. It demonstrates in vitro activity against MSSA and MRSA; however, its effectiveness can be reduced in thymidine-rich environments, such as damaged tissues, potentially limiting its role in endocarditis [7]. Clinical data suggest that oral TMP/SMX, particularly when combined with clindamycin, can serve as step-down therapy for *S. aureus* endocarditis in selected patients, achieving outcomes comparable to continued intravenous therapy [31].

Scientific evidence mostly supports the use of dual oral OAT to optimize bactericidal activity and prevent relapse, which mirrors the rationale of IV therapy principles for certain organisms. These data, particularly PK/PD principles, explain why specific antibiotic classes are prioritized in clinical studies of OAT for IE. A summary of the bioavailability, PK/PD considerations, MIC-related limitations, and IE-specific evidence for each oral agent is provided in Table 1.

## 3. Review of Clinical Evidence Supporting Oral Therapy

### 3.1. Randomized Controlled Trials

The POET trial was the first large RCT to evaluate early transition from IV to OAT in stable patients with left-sided IE. In this randomized, noninferiority, multicenter trial, 400 adults with left-sided IE caused by *Streptococcus* spp., *Enterococcus faecalis*, MSSA, or coagulase-negative staphylococci were enrolled [6]. Patients achieved a clinically stable condition after IV antibiotic was administered for at least 10 days prior to randomization [6]. This ensured clinical improvement before intervening. The primary composite outcome (all-cause mortality, unplanned cardiac surgery, embolic events, or relapse of bacteremia with the primary pathogen, from the time of randomization until 6 months after antibiotic treatment was completed) was observed in 12.1% of the IV-treated group (*n* = 199) versus 9% of the orally treated group (*n* = 201), thus meeting noninferiority criteria [6]. Adverse events occurred in 56% of patients in each group, mainly mild gastrointestinal reactions, rash, or transient liver enzyme elevation. Serious events, including heart failure, embolic episodes, unplanned cardiac surgery, relapse, and death, were comparable, with 24% in the step-down oral arm versus 26% in the IV arm, while catheter-related complications occurred in 3% of patients receiving IV therapy and in none receiving oral therapy. Median hospitalization duration post randomization was 19 days for patients in the IV arm versus 3 days for patients in the oral arm (*p* < 0.001) [6].

In a POET sub-study, 368 patients with larger vegetation size (≥10 mm) and early cardiac surgery without aortic root abscess were followed for a median of 1406 days [32]. Step-down OAT was noninferior to continued IV treatment in all subgroups, confirming its safety even in select high-risk patients [32].

The POET PK/PD sub-study demonstrated that most oral regimens achieved predefined target antibiotic levels (PTAs), with high probabilities of target attainment for amoxicillin, linezolid, moxifloxacin, and rifampicin, though markedly lower PTA for dicloxacillin [15]. Importantly, patients with sub-target levels to one agent were adequately compensated by dual–drug regimens [15].

Moreover, follow-up analysis at both 3.5 and 5 years confirmed the original hypothesis that step-down OAT therapy is noninferior to continued IV antibiotic administration [33,34]. Also, in the 5-year post hoc follow-up, after a median of 5.4 years, patients receiving step-down oral therapy had a lower incidence of the primary composite outcome than those in the IV-treated group (HR, 0.65; 95% confidence interval [CI], 0.47 to 0.90) [34]. The difference was attributed to reduced all-cause mortality in the oral group, while rates of unplanned surgery, embolic events, and relapse of bacteremia remain similar [34]. No long-term treatment failures were observed, and findings were consistent across prespecified subgroups [34].

Currently ongoing randomized trials, RODEO-1 and RODEO-2, are assessing the safety and efficacy of switching from IV to OAT in left-sided IE due to staphylococci, streptococci, or enterococci, to define optimal regimens and selection criteria [14].

### 3.2. Real-World Evidence

Following the landmark POET trial in 2018, several observational studies have evaluated the feasibility and outcomes of oral step-down therapy in routine clinical practice.

A 2019 multicenter retrospective cohort from France included 344 patients with left-sided *S. aureus* IE, of whom 84 were transitioned to high-dose TMP-SMX (two double-strength tablets three times daily) with or without clindamycin at 1800 mg per day. Compared with the patients who completed the full course of IV therapy, the oral group had high cure rates with few relapses and showed significantly lower in-hospital mortality (10% vs. 18%) and overall mortality (19% vs. 30%). The findings were both clinically and statistically significant [31].

The 2021 Dutch retrospective analysis evaluated patient eligibility for oral therapy using POET criteria. They estimated that one-third of patients, mainly those with *Streptococcus* spp. or *E. faecalis* IE, could have transitioned safely after 2 or 3 weeks of IV therapy and likely completed treatment without a clinically significant difference in outcomes [35].

The 2023 Danish POETry study included 562 patients, most of whom had left-sided native-valve IE. 43% were transitioned to OAT after 10–21 days of IV treatment. The most frequently isolated pathogens were *Streptococcus* spp., *E. faecalis*, and *S. aureus*, excluding MRSA. In line with the POET protocol, 95% received dual oral regimens with high bioavailability, typically a β-lactam combined with a fluoroquinolone, linezolid, or rifampin. Adverse events after switching to oral therapy were infrequent, as only 6% of the patients had mild gastrointestinal or cutaneous reactions. The primary composite outcome (death, unplanned cardiac surgery, embolic events, or relapse within 6 months) occurred in 9.4% of the oral group versus 13.1% of the IV group, a difference that was neither clinically nor statistically significant [36].

A 2023 multicenter study from the United States included 257 patients, nearly all with left-sided IE, 46 of whom were transitioned to OAT after clinical stabilization. The most common oral regimen used was linezolid 600 mg twice daily, used in 65% of patients; among these, 87% received linezolid alone, and 13% received linezolid combined with rifampin. High-dose oral β-lactams (ampicillin, amoxicillin, dicloxacillin, or penicillin V) were used in 17% of the cases, while other oral regimens included fluoroquinolone + rifampin or TMP/SMX ± rifampin. Targeted pathogens were mainly *S. aureus*, *E. faecalis*, and viridans-group streptococci. When compared with patients who remained on IV therapy, the oral group demonstrated similar 90-day clinical success (91% vs. 88%) and similar mortality (4% vs. 5%), but experienced fewer line-related complications (0% vs. 13%) and fewer adverse drug events (9% vs. 18%), most commonly cytopenias or gastrointestinal intolerance. Smaller studies have shown consistent findings [37].

In a 2024 case series from Milan, nine patients transitioned to OAT after 10–21 days of IV antibiotics, most receiving linezolid 600 mg twice daily or fluoroquinolone-based combinations targeting *S. aureus*, *E. faecalis*, and *Streptococcus* spp. No relapses, deaths, or adverse drug reactions were reported, and although clinically encouraging, the results did not reach statistical significance due to the small sample size [38].

### 3.3. Systematic Reviews and Meta-Analysis

A number of systematic reviews and meta-analyses have been published in the past few years that have examined the role of OAT in IE. A 2022 systematic review by Wald-Dickler et al. investigated RCTs comparing oral and IV treatment for serious infections, among which three RCTs focused on IE. Two of them demonstrated noninferiority, and the largest trial confirmed the superiority of OAT with lower mortality and relapse rates. The overall meta-analysis was significant for an 8% higher treatment success rate in OAT as compared to the standard IV regimens [39].

In a more recent systematic review and meta-analysis published in 2024, which specifically focused on IE, 6 out of 29 studies met the inclusion criteria. They involved 840 IV-treated and 677 patients treated with OAT. The meta-analysis showed that step-down OAT in clinically stable patients did not lead to higher rates of treatment failure, complications, or death. Remarkably, the rate of recurrence in the oral switch group was significantly lower (OR 0.54, 95% CI 0.31–0.92). Even though the hospital stay was mildly reduced with OAT, the result was not statistically significant. Noninferiority was confirmed by a subgroup analysis including only RCTs; however, a wide confidence interval was noted, indicating variability and a small sample size. The authors concluded that transitioning from IV to OAT appears to be effective in selected patients with IE [40].

Finally, a more recent systematic review and meta-analysis of RCTs, published in 2025, evaluated oral versus IV therapy only for *S. aureus* IE and bacteremia. Their comprehensive search identified 4 RCTs meeting strict inclusion criteria, including 204 patients who received OAT and 186 treated with an IV regimen. The combined analysis revealed no difference in therapeutic failure between the two groups (RR 0.99; 95% CI 0.63–1.57). Moreover, both oral and IV treatment groups had similar adverse event rates (RR 0.65; 95% CI 0.07–5.94). However, this estimate was limited by heterogeneity and wide confidence intervals. Notably, most participants were stable and had uncomplicated disease with limited representation of MRSA across the trials [41].

Overall, the cumulative evidence validates the efficacy and safety of oral treatment in carefully selected patients with IE while highlighting the importance of conducting larger RCTs. In this context, “carefully selected patients” refers to those with IE who are clinically stable, improving on therapy, free of major complications on imaging, and able to reliably absorb and adhere to oral medications.

## 4. Practical Implementation

**Figure 1:**
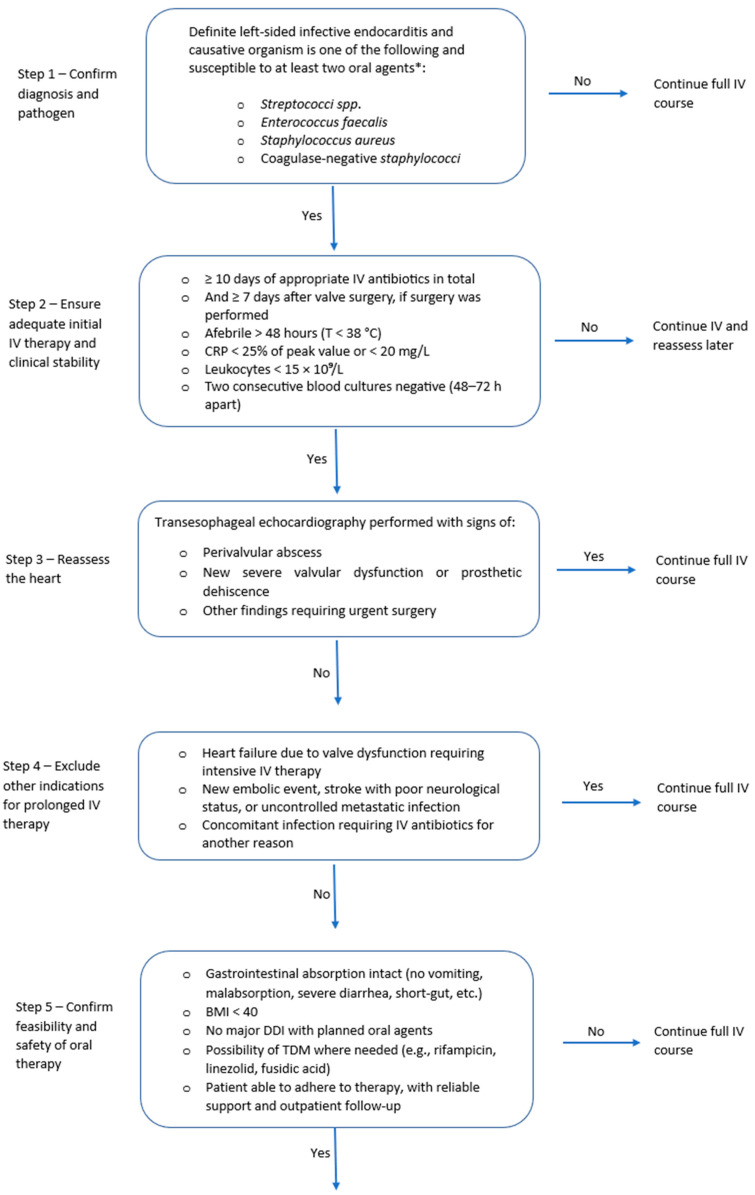

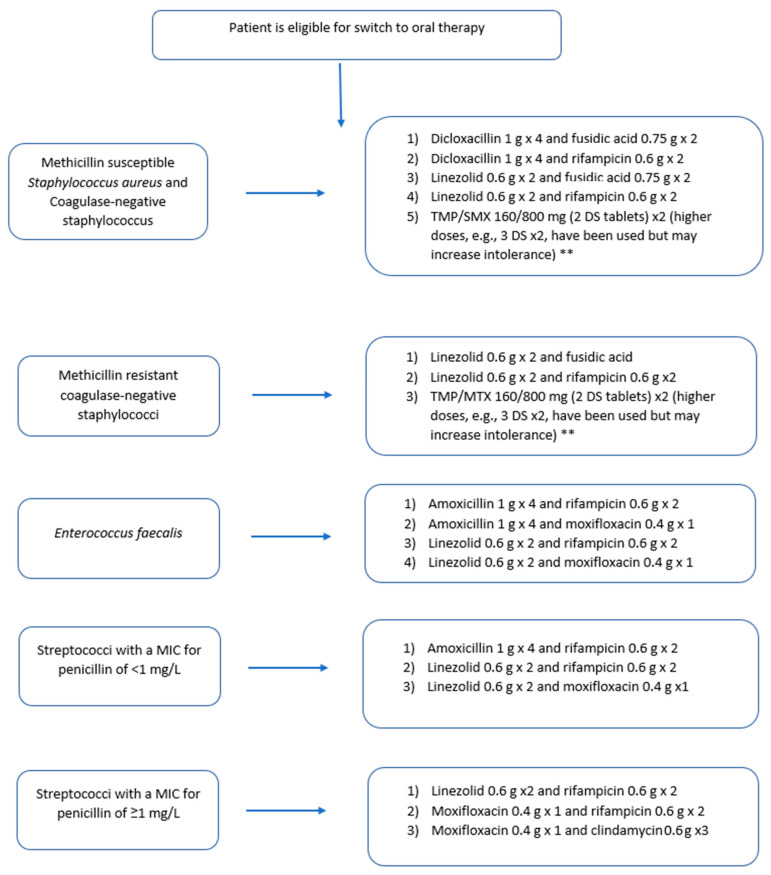
Algorithm for switching from IV to oral antibiotic therapy in left-sided infective endocarditis.

Synthesizing the available studies, the following framework can guide the implementation of OAT in IE (Figure 1): Eligible candidates are adults with definite left-sided IE due to MSSA, coagulase-negative staphylococci, viridans-group *Streptococcus* spp., or *E. faecalis* who have completed at least 10 days of effective IV treatment (or ≥7 days after valve surgery); are afebrile for more than 48 h; show biochemical improvement; have no abscess or new surgical indication on recent transesophageal echocardiography (TOE); and have no barriers to oral absorption or adherence to therapy [6]. When switching to OAT, it is advisable to have predefined PK/PD targets for the chosen oral agents and consider an early exposure check (e.g., through TDM) to confirm efficacy. Oral combination therapy is preferentially prescribed. A promising combination for *E. faecalis* would be amoxicillin combined with cefditoren when switching from an IV ampicillin–ceftriaxone regimen, as demonstrated by a case series of prosthetic valve endocarditis (PVE) by Attanasio et al. [42]. However, this strategy requires confirmation in further studies.

When selecting a combination therapy, drug–drug interactions and potential antagonism must be considered. Rifampicin is a potent inducer, and reductions in blood levels of co-administered drugs have been documented with moxifloxacin, clindamycin, and linezolid [43,44]. Although linezolid–rifampicin has been used successfully in the treatment of *Mycobacterium tuberculosis*, this PK behavior should not be extrapolated to IE, as *M. tuberculosis* is far more susceptible to linezolid than staphylococci. Moreover, caution warranted with staphylococcal β-lactam (e.g., dicloxacillin), as a PK/PD analysis based on TDM data from the POET trial showed very low PTA (9–17%) for dicloxacillin against staphylococci [15]. After the switch, the OPAT protocol can be adapted for OAT in terms of patient education on daily temperature and symptom recording; frequent nursing contact; and scheduled physician review at least weekly. Laboratory safety monitoring remains essential for certain drugs, particularly weekly complete blood counts with linezolid and periodic liver enzyme assessment with rifampicin; electrocardiography is needed with agents that are known to cause QT-prolongation, such as quinolones.

Special clinical contexts require additional consideration. Clinically stable PVE without a surgical indication may be considered for OAT step-down, often incorporating rifampicin for staphylococcal infection due to its ability to penetrate biofilms. In device-related IE, infected hardware should be removed before switching to oral therapy is contemplated. An early study investigating OAT in IV drug users suggested that OAT may be used upon discharge, including in right-sided IE, as maintaining IV access is not advised in this group of patients [5]. Another study by Dworkin et al. reported that 14 patients with right-sided IE secondary to MSSA were successfully switched to ciprofloxacin and rifampicin after 7 days of IV therapy, with no adverse events or relapse of infection [45]. Although additional evidence is needed to guide management in these clinical scenarios, existing data support the potential switch to OAT with a selected individualized approach. For MRSA, Gram-negative infections, complicated IE, or in pediatric patients, evidence remains insufficient; IV therapy remains the standard of care, and any oral use should be carefully individualized weighing advantages and disadvantages.

## 5. Guidelines

Both the American and European guidelines for IE mention oral linezolid as an option for a first-line regimen for enterococcal endocarditis resistant to ampicillin and vancomycin. In addition, both mention oral ciprofloxacin as an alternative treatment for HACEK (*Haemophilus* spp., *Aggregatibacter* spp., *Cardiobacterium hominis*, *Eikenella corrodens*, and *Kingella* spp.) endocarditis [7,46]. Following the POET trial, the recent European Society of Cardiology (ESC) guidelines adopted the regimens of the trial as options for step-down therapy for IE, provided all criteria mentioned in the study are fulfilled [46]. The ESC guidelines also include antibiotics not tested thoroughly in the POET study, like cefditoren + amoxicillin for *E. faecalis* endocarditis or linezolid + rifampicin for MSSA and enterococcus or moxifloxacin + rifampicin or combination with fuscidic acid 750 mg for the organisms mentioned in the POET study [11]. There are currently no new updates from the American Heart Association (AHA) or the Infectious Diseases Society of America (IDSA) regarding step-down OAT in IE.

## 6. Challenges, Gaps, and Ongoing Trials

Despite the encouraging data regarding the safety and efficacy of step-down OAT in IE, many areas remain uncertain. It should be first noted that in the clinical studies, all patients transitioned to OAT only once they were clinically stable with no complications related to IE. In real-world practice, many patients do not meet these criteria, as clinical stability varies considerably, making routine transition to OAT more challenging. Additionally, outcomes definitions differed across studies: mortality was assessed at different time intervals, and the criteria of “cure” and “relapse” were not standardized. Another limitation is the inconsistency in the duration of IV therapy prior to transitioning to OAT. It is not yet clear whether a fixed period of IV therapy is necessary for all patients or whether an individualized approach guided by clinical response and PK/PD targets would be equally appropriate. Similarly, the most appropriate antibiotic classes, the role of monotherapy versus combination therapy, and guidelines about the correct dosing, particularly in relation to body weight, hepatic or renal impairment, and use of TDM, still require delineation. Moreover, evidence is lacking in special populations like patients with IE due to MRSA and Gram-negative infections, as well as pediatric patients. Thus, future trials must address all remaining unanswered questions and investigate the ideal timing of transition, the choice and dosing of OAT, and its use in specific population groups.

## 7. Conclusions

Transitioning from IV to OAT represents a promising and evidence-based strategy for the management of IE in appropriately selected patients, i.e., in cases where clinical stability is established, the causative pathogen is susceptible, and antibiotics with high bioavailability are used. Implementation requires careful adherence to selection criteria, attention to PK/PD targets, and regular clinical and laboratory monitoring. While ESC guidelines have adopted this approach following the POET trial, significant knowledge gaps persist, including the ideal timing of transition, dosing optimization, and validation in special populations such as patients with MRSA, Gram-negative, and pediatric infections. Further large-scale studies are warranted to refine these parameters and standardize protocols.

## Figures and Tables

**Table 1:** Pharmacokinetic/pharmacodynamic characteristics, bioavailability, and evidence summary for oral antibiotic options in infective endocarditis.

Drug/Class	Bioavailability	Key PK/PD Considerations	MIC/Exposure Limitations	Evidence/Notes for IE
Amoxicillin	Dose-dependent; 100% at 375 mg → 55% at 3000 mg	Saturable absorption	Effective only if MIC ≤ 2 mg/L at standard 1 g TID; doses ≥ 2.5 g TID needed for MIC > 8 mg/L	Used for streptococci and *E. faecalis*; high-dose regimens under evaluation (RODEO-2)
Amoxicillin–clavulanate	Similar to amoxicillin	Similar to amoxicillin	Requires MIC ≤ 1–2 mg/L to achieve targets	Limited IE data
Semi-synthetic penicillins (dicloxacillin, oxacillin, cloxacillin)	Sub-optimal, e.g., Dicloxacillin 38–50%; oxacillin ~30%	Food reduces absorption by >40%; short half-life → frequent dosing needed (q4–6h)	Drug concentrations should remain above the MIC for 60–70% of the dosing interval	Standard IV agents for MSSA; oral forms have limited evidence in IE
Fluoroquinolones	High for most agents	Concentration-dependent (AUC/MIC)	Moxifloxacin AUC/MIC effective but resistance risk if MIC > 0.06–0.125 mg/L; delafloxacin MIC_50_ 0.004 µg/mL but no IE studies	Ciprofloxacin + rifampin effective for right-sided MSSA IE in PWID; limited streptococcal efficacy in models
Linezolid	~100%	AUC/MIC-driven; significant interindividual variability	Toxicity risk at high exposure; reduced efficacy if MIC ≥ 2–4 mg/L	Case series support use as salvage or step-down; requires TDM in renal/hepatic dysfunction
Rifampicin	Excellent	Potent inducer; biofilm penetration	Markedly reduces clindamycin exposure	Useful in prosthetic material IE; only in combination
Clindamycin	~60% but reduced to 15% with rifampicin	Dosing should be adjusted according to body weight and drug clearance	Major interaction with rifampicin	No IE studies
TMP/SMX	Up to 90%	Concentration-dependent; synergistic mechanism	Efficacy may be reduced in thymidine-rich tissues	Effective for MSSA/MRSA in high-dose regimens; oral step-down success shown in cohort studies

PK/PD: pharmacokinetics and pharmacodynamics; MIC: minimum inhibitory concentration; IE: infective endocarditis; IV: intravenous; MSSA: methicillin-susceptible *Staphylococcus aureus*; AUC: area under the curve; PWID: people who inject drugs; q4–6h: Every 4 to 6 h; TDM: therapeutic drug monitoring; TID: three times daily; TMP/SMX: trimethoprim–sulfamethoxazole; MRSA: methicillin-resistant *S. aureus*.

## Data Availability

No new data were created or analyzed in this study. Data sharing is not applicable to this article.

## Abbreviations

IE	Infective endocarditis
IV	Intravenous
OPAT	Outpatient IV antimicrobial therapy
PK/PD	Pharmacokinetics and pharmacokinetics
RCT	Randomized controlled trial
T>MIC	Time above the minimal inhibitory concentration
AUC	Area under the curve
MSSA	Methicillin-susceptible *Staphylococcus aureus*
TDM	Therapeutic drug monitoring
TMP/SMX	Thrimethoprim–sulfamethoxazole
PTA	Predefined target antibiotic level
TOE	Transesophageal echocardiography
PVE	Prosthetic valve endocarditis
ESC	European Society of Cardiology
AHA	American Heart Association
IDSA	Infectious Diseases Society of America
